# Comparison of 1-year clinical outcomes between prasugrel and ticagrelor versus clopidogrel in type 2 diabetes patients with acute myocardial infarction underwent successful percutaneous coronary intervention

**DOI:** 10.1097/MD.0000000000014833

**Published:** 2019-03-15

**Authors:** Kye Taek Ahn, Seok-Woo Seong, Ung Lim Choi, Seon-Ah Jin, Jun Hyung Kim, Jae-Hwan Lee, Si Wan Choi, Myung Ho Jeong, Shung Chull Chae, Young Jo Kim, Chong Jin Kim, Hyo-Soo Kim, Myeong-Chan Cho, Hyeon-Cheol Gwon, Jin-Ok Jeong, In-Whan Seong

**Affiliations:** a Division of Cardiology, Department of Internal Medicine, Chungnam National University Hospital, Chungnam National University, School of Medicine, Daejeon; b Chonnam National University Hospital, Chonnam National University, School of Medicine, Gwangju; c Kyungpook National University Hospital, Kyungpook National University, School of Medicine; d Yeungnam University Hospital, Daegu; e Kyunghee University College of Medicine; f Seoul National University Hospital, Seoul National University, School of Medicine, Seoul; g Chungbuk National University Hospital, Chungbuk National University, School of Medicine, Cheongju; h Samsung Medical Center, Sungkyunkwan University, School of Medicine, Seoul, Republic of Korea.

**Keywords:** diabetes, myocardial infarction, prasugrel, revascularization, ticagrelor

## Abstract

Although the new oral P2Y_12_ inhibitors, prasugrel/ticagrelor have shown greater efficacy than clopidogrel in patients with the acute coronary syndrome, but they have not shown better efficacy in Korean patients. So we evaluated the efficacy of the prasugrel/ticagrelor in patients with myocardial infarction (MI) and diabetes, a more high-risk patients group.

From the Korea Acute Myocardial Infarction Registry-National Institute of Health, 3985 patients with MI and diabetes who underwent PCI were enrolled between November 2011 and December 2015. The patients were divided into 2 groups: clopidogrel (n = 2985) and prasugrel/ticagrelor (n = 1000).

After propensity score matching, prasugrel/ticagrelor group showed a no significant difference in risk of the composite of cardiac death (CD), recurrent MI or stroke (hazard ratio [HR], 0.705; 95% confidence interval [CI], 0.474–1.048; *P* = .084). However, the risk of major bleeding was significantly higher in the prasugrel/ticagrelor group. (HR; 2.114, 95% CI; [1.027–4.353], *P* = .042). In subgroup analysis, major bleeding was significantly increased in the subgroup of creatinine clearance <60 ml/min/1.73 m^2^, hypertension, underwent a trans-femoral approach and diagnosed as NSTEMI among the prasugrel/ticagrelor group.

The use of prasugrel/ticagrelor did not improve the composite of CD, recurrent MI or stroke, however, significantly increased major bleeding events in Korean patients with MI and diabetes undergoing PCI.

## Abstract 2

Supplemental Digital Content is available in the text

## 1 Introduction

Although the interventional technique and medical treatment were improved for decades, thrombotic events still remain the serious cause of mortality after percutaneous coronary interventions (PCI). New oral P2Y_12_ inhibitors, prasugrel/ticagrelor are shown greater antiplatelet efficacy than clopidogrel; recent guideline recommended their use in patients with acute myocardial infarction (AMI).^[1,2]^ However, these agents have been associated with higher bleeding risk and reported increased response especially in East Asian patients.^[3–8]^

Prasugrel/ticagrelor would be the preferred treatment option in type 2 diabetes mellitus patients with AMI undergoing PCI, but there is no convincing evidence that the presence of diabetes should affect decision making with respect to the choice of them.^[9]^ Moreover, there were little data about the clinical impact of prasugrel/ticagrelor compared with clopidogrel in Korean patients with MI and diabetes. Therefore, we compared the 1-year clinical outcomes between prasugrel/ticagrelor and clopidogrel in patients with MI and diabetes underwent PCI.

## 2 Materials and methods

### 2.1 Study design and population

We consecutively selected patients with MI and diabetes mellitus who underwent successful PCI from the database of the Korea Acute Myocardial Infarction Registry-National Institutes of Health (KAMIR-NIH). The KAMIR-NIH is a prospective, multicenter, web-based observational cohort study to develop the prognostic and surveillance index of Korean patients with AMI from 20 centers in Korea and has been performed to support by a grant of Korea Centers for Disease Control and Prevention from November 2011 to December 2015.

**Figure 1:**
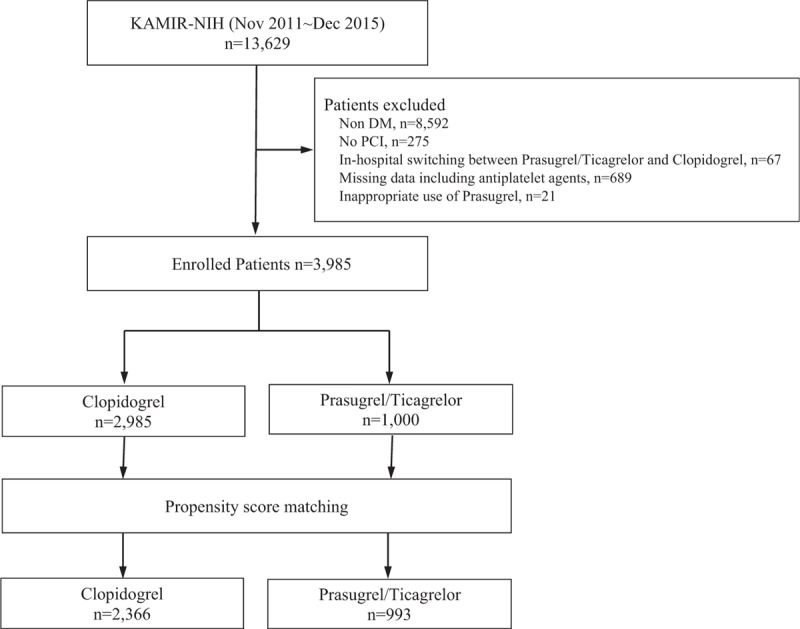
Study population flow chart. The patients with MI and diabetes who underwent PCI were enrolled. We excluded the patients occurred in-hospital switching between antiplatelet agents, those inappropriately used prasugrel, or those had missing data about antiplatelet agents or clinical outcomes. Total 3985 patients were enrolled. Among them, 2985 patients included clopidogrel group and 1000 patients included the prasugrel/ticagrelor group. MI = myocardial infarction, PCI = percutaneous coronary intervention.

The diagnosis of AMI was based on detection of a raise and/or fall of cardiac biomarker (creatinine kinase-muscle/brain [CK-MB] and troponin I or T) with at least 1 value above the 99th percentile upper reference limit and with at least 1 of the following: symptoms of ischemia, new or presumed new significant ST- segment or T - wave changes or new left bundle branch block, development of pathological Q waves in the ECG, and imaging evidence of new loss of viable myocardium or new regional wall motion abnormality. Among them, we excluded the patients who discontinued antiplatelet agents during hospitalization or those occurred in-hospital switching between clopidogrel, prasugrel, and ticagrelor. Patients with unappropriated use of prasugrel were also excluded (age ≥75, body weight <60 kg, history of transient ischemic attack (TIA) or stroke), (Fig. 1). The study protocols were approved by the ethics committee at each participating centers and followed the principles of the Declaration of Helsinki. All patients provided written, informed consent for participation in the registry. Trained study coordinators at each participating institution collected the data using a standardized format. Standardized definitions of all variables were determined by the steering committee board of KAMIR-NIH.

### 2.2 Interventional procedures and in-hospital medications

The choice of antiplatelet agents (clopidogrel or ticagrelor or prasugrel), emergent or early invasive treatments strategies, predilatation or postdilatation, type of stents, use of periprocedural glycoprotein IIb/IIIa inhibitors, and antithrombotic medication were determined based on the clinical status of AMI patient according to the clinical decision of operators in each institutes. PCI was performed in a routine manner. Anti-platelet agents were administered to all patients prior to the intervention, with aspirin 300 mg loading dose (LD) and clopidogrel 300–600 mg LD or ticagrelor 180 mg LD or prasugrel 60 mg LD. After the intervention, the patients received aspirin 100 mg once daily indefinitely and clopidogrel 75 mg once daily or ticagrelor 90 mg twice daily or prasugrel 10 mg once daily for at least 1 year. Other medical treatments were also used based on the standard treatment regimen for patients with AMI in a nonrestrictive manner.

### 2.3 Study endpoint

The primary efficacy end-point was a composite of cardiac death (CD), recurrent MI or stroke at 1-year. The safety end-point was major bleeding at 1-year. The CD was defined as death from arrhythmia, pump failure, or mechanical complications including free wall rupture and ventricular septal rupture. Definition of major bleeding is as follows:

(1) ≥5 g/dl hemoglobin (Hb) decrease or,
(2) ≥15% of hematocrit (Hct) decrease or,
(3) presence of intracranial hemorrhage (ICH).

### 2.4 Statistical analysis

Continuous variables were expressed as mean ± standard deviation or the median and interquartile range, and they were compared using the independent *t* test or Mann–Whitney U test between the 2 groups. Categorical variables were compared with Pearson Chi-square or Fisher exact tests between the 2 groups. To minimalize the effect of selection bias in the direct comparison between ticagrelor or prasugrel and clopidogrel, the propensity score was estimated using a multivariable logistic regression model, in which treatment status is regressed on the observed baseline, clinical, angiographic, and procedural characteristics. We performed multiple imputation procedure to fill in for missing data of several important variables such as left ventricular ejection fraction, initial systolic blood pressure and initial heart rate. Model discrimination was measured by the c-static and calibration was assessed by the Hosmer–Lemeshow goodness-of-fit test (c-statistic: 0.647, Hosmer–Lemeshow: *P* = .169). The results of the multivariable models were verified using a propensity score matching method. Thereafter, the patients receiving clopidogrel were 1-to-N matched to the patients receiving ticagrelor or prasugrel on propensity scores using the nearest available pair matching method except for the subject including missing values.^[10]^ In the propensity score-matched populations, the baseline clinical, angiographic, and procedural covariates were compared between the 2 groups. Multivariate Cox regression analysis with an “enter” method was used to identify the independent predictors of clinical outcomes. Only variables with a *P*-value < .05 in the univariate analysis were included in the multivariate model. All statistical tests were 2-tailed, and a *P*-value < .05 was considered statistically significant. All the statistical analyses were performed using SPSS (Statistical Package for Social Science, SPSS Inc, Chicago, IL) for Windows, Version 21.0.

## 3 Results

### 3.1 Baseline characteristics

A total of 3985 patients were enrolled in the current study between November 2011 and December 2015. The average follow-up day was 351. Enrolled patients were divided into 2 groups: clopidogrel group (n = 2985) and prasugrel/ticagrelor group (prasugrel = 351/ ticagrelor = 649, total n = 1000).

Baseline characteristics of patients before and after propensity score matching were presented in Table 1. Before propensity score matching, the baseline characteristics were significantly different between the clopidogrel and the prasugrel/ticagrelor group. Patients of the clopidogrel group were older and had a lower body mass index (BMI). The patients with hypertension, dyslipidemia, previous MI, previous stroke were more likely to the clopidogrel group. In laboratory data, creatinine clearance (Ccr), low-density lipoprotein, CK-MB level were significantly higher in the prasugrel/ticagrelor group. In hemodynamic and procedural characteristics, more patients of the prasugrel/ticagrelor group were with lower Killip class, symptom to balloon time and heart rate at admission. Prasugrel/ticagrelor group had more left anterior descending artery disease as the infarct-related vessel. More culprit lesion PCI was done in the prasugrel/ticagrelor group. Glycoprotein IIb/IIIa inhibitor, statin, beta-blocker, and oral hypoglycemic agent were more used in the prasugrel/ticagrelor group than the clopidogrel group. After propensity-score matching, there were less significant differences in baseline characteristics. However, BMI, symptom to balloon time, level of Hb, Ccr, CK-MB were higher in the prasugrel/ticagrelor group. More transfemoral vascular approaches were done in the clopidogrel group. Glycoprotein IIb/IIIa inhibitor was more prescribed in the prasugrel/ticagrelor group than the clopidogrel group during hospitalization.

**Table 1:**
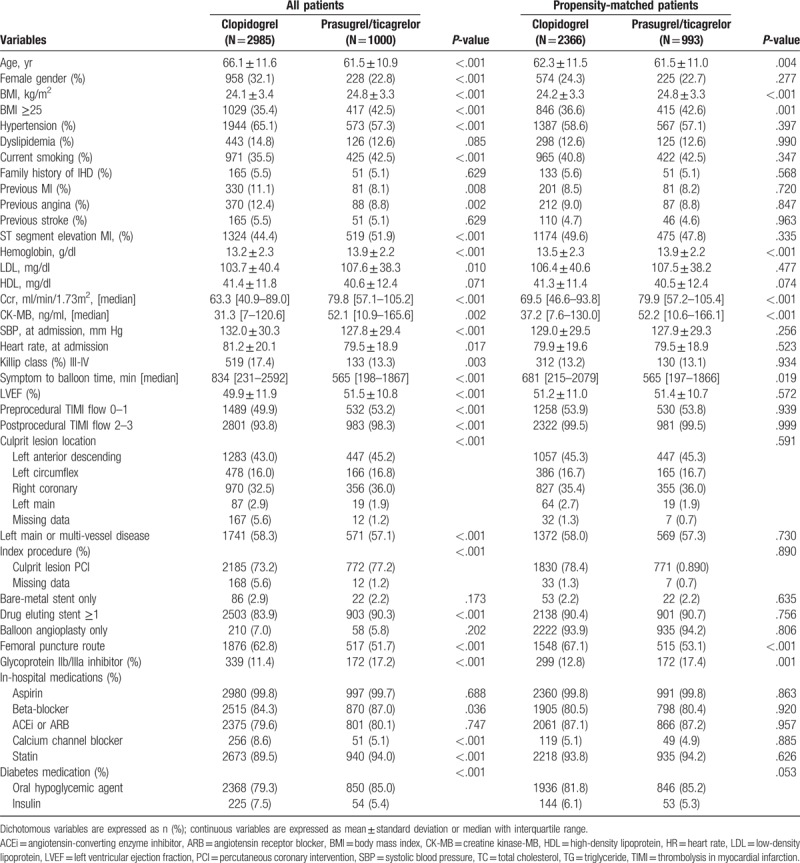
Baseline clinical, procedural, medical characteristics between the clopidogrel and the prasugrel/ticagrelor group before and after propensity score matching.

### 3.2 Clinical outcomes in all patients

**Figure 2:**
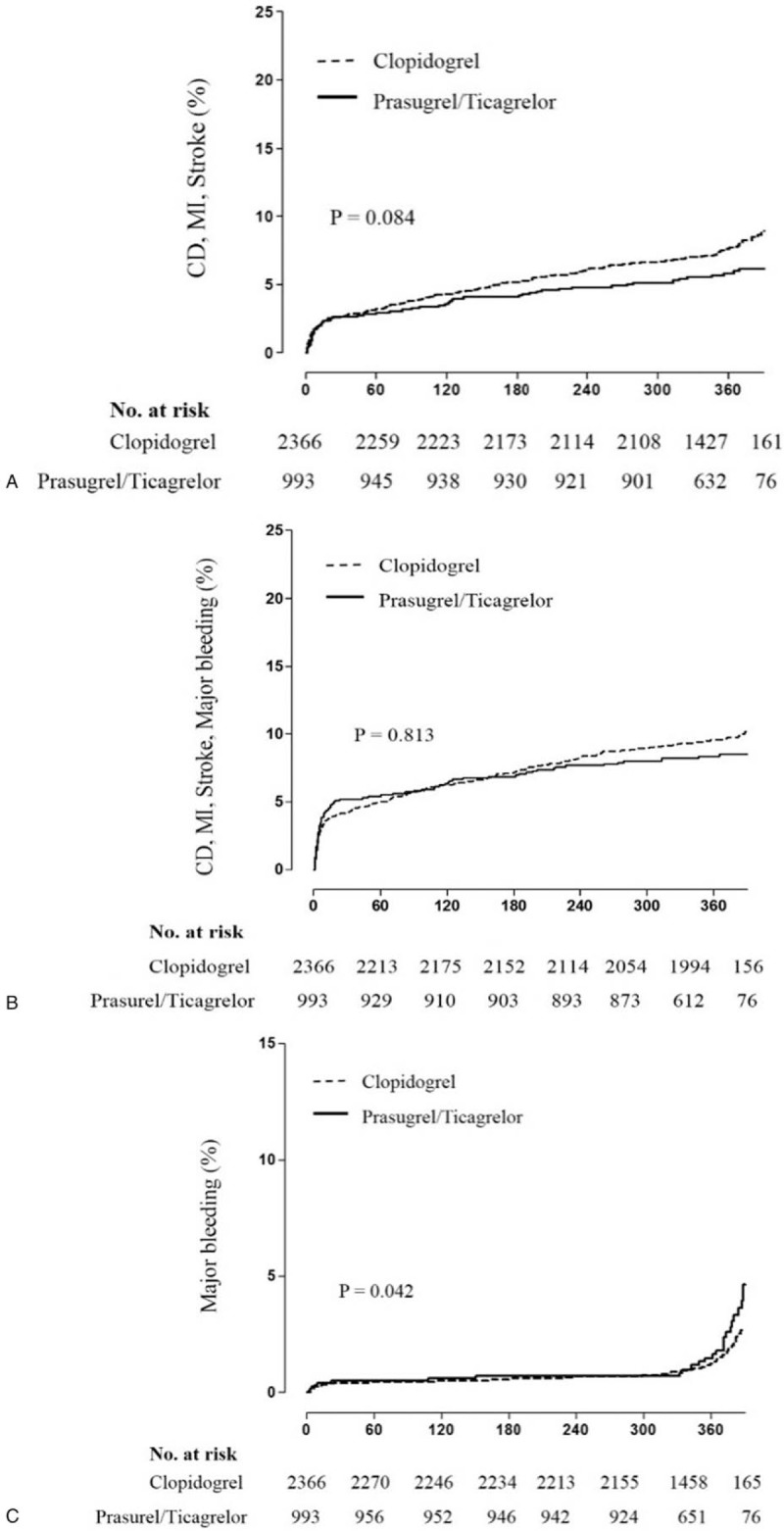
Kaplan–Meier survival curve between clopidogrel and prasugrel/ticagrelor group in the propensity-matched cohort. (A) the composite of CD, recurrent MI or stroke, (B) the composite of CD, recurrent MI, stroke or major bleeding, (C) Major bleeding. The Kaplan–Meier curves presented the event rates for 1-year between clopidogrel and prasugrel/ticagrelor groups in the propensity-matched cohort. (A) showed the composite of CD, recurrent MI or stroke. (B) showed the composite of CD, recurrent MI, stroke or major bleeding. (C) showed major bleeding. There was no significant difference in the risk of the composite of CD, recurrent MI or stroke between 2 groups. However, the risk of the major bleeding was significantly higher in the prasugrel/ticagrelor group in the multivariate Cox regression analysis. CD = cardiac death, MI = myocardial infarction.

The details of unadjusted and adjusted 30-day and 1-year clinical outcomes before and after propensity score matching were shown in Table 2. The composite of CD, recurrent MI or stroke were occurred in 136 (3.4%) and 392 (9.8%) patients during 30-day and 1-year follow-up. In multivariate Cox regression analysis, there was no significant difference in the risk of the composite of CD, recurrent MI or stroke between the clopidogrel and the prasugrel/ticagrelor group at 30-day and 1-year. After propensity-score matching, there was also no significant difference in the risk of the composite of CD, recurrent MI or stroke between the 2 groups. Major bleeding events were occurred in 23 (0.6%) and 93 (2.3%) patients during 30-day and 1-year follow-up. In the clopidogrel group, major bleeding events occurred in 65 (2.2%) of 32 cases of ≥5 g/dl Hb decrease, 16 cases of ≥15% Hct decrease and 17 cases of ICH. In the prasugrel/ticagrelor group, major bleeding events occurred in 28 (2.6%) of 14 cases of ≥5 g/dl Hb decrease, 12 cases of ≥15% Hct decrease, and 2 cases of ICH. The adjusted hazard ratio (HR) of the major bleeding was significantly higher in the prasugrel/ticagrelor group (HR; 2.114, 95% confidence interval [CI]; [1.027–4.353], *P* = .042). We analyzed the clinical impact of clopidogrel and prasugrel/ticagrelor in various subgroups. Especially, major bleeding was significantly increased in the subgroup of Ccr <60 ml/min/1.73 m^2^, hypertension, underwent a trans-femoral approach and diagnosed as NSTEMI among the prasugrel/ticagrelor group. Other results of detailed subgroup analyses are provided in the supplemental appendix. Figure 2 shows the Kaplan–Meier curves for 1-year clinical outcomes between the clopidogrel and the prasugrel/ticagrelor group in the propensity-matched cohort.

**Table 2:**
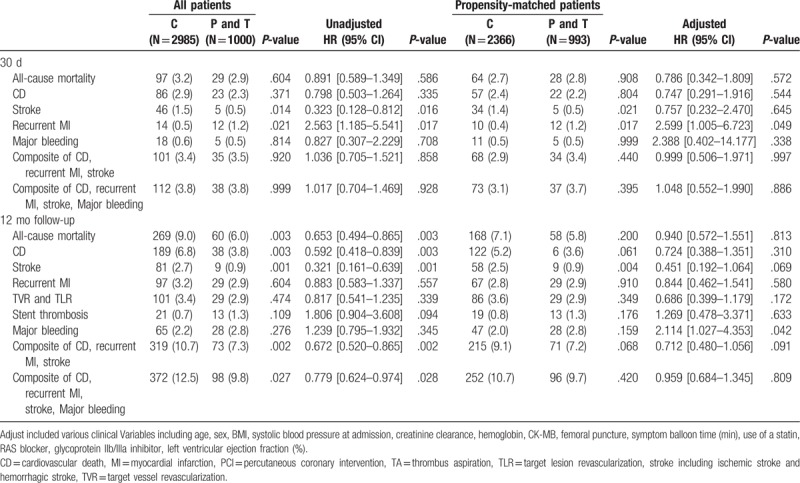
Clinical outcomes between the clopidogrel and the prasugrel/ticagrelor group before and after propensity score matching.

We compared the clinical outcomes in patients with HbA1C >6.5 (Table 3). The primary end-points and safety end-points were not significantly different between the clopidogrel and the prasugrel/ticagrelor group.

**Table 3:**
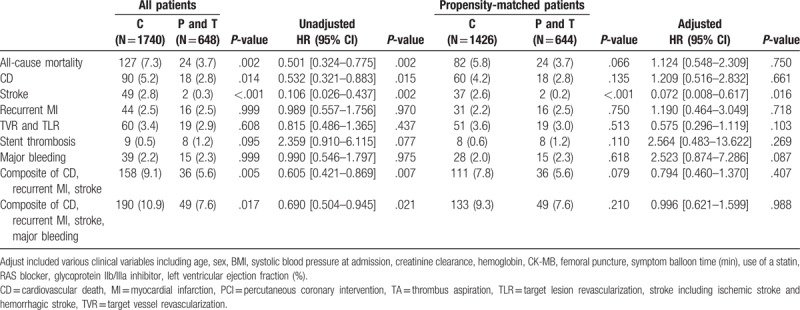
Clinical outcomes between the clopidogrel and the prasugrel/ticagrelor group before and after propensity score matching, HbA1C >6.5.

## 4 Discussion

We conducted this study to compare the 1-year clinical outcomes between the clopidogrel and the prasugrel/ticagrelor group in patients with MI and diabetes undergoing PCI. The main findings of our study were that the prasugrel/ticagrelor did not improve the primary end-points (composite of CD, recurrent MI or stroke), but increased major bleeding events in patients with MI and diabetes undergoing PCI. Moreover, the bleeding event increased in patients with low GFR (Ccr <60 ml/min/1.73 m^2^), with hypertension, underwent a trans-femoral approach and diagnosed as NSTEMI.

Diabetes is highly associated with accelerated atherothrombosis. Patients with diabetes have had a more than 2-fold risk of coronary heart disease and stroke, and other vascular cause death.^[11,12]^ Several mechanisms are demonstrated the prothrombotic state of diabetes; diabetes induces the platelet dysfunction by hyperglycemia, insulin resistance, upregulation of GP IIb/IIIa expression, P2Y_12_ signaling, increased platelet turnover, and excessive oxidative stress.^[13–15]^ Therefore, acute coronary syndrome (ACS) patients with diabetes have a high thrombotic risk and need antiplatelet combination strategies involves pathways TXA2 and ADP-P2Y_12._

Numerous data have demonstrated a close relationship between low response to clopidogrel and atherothrombotic events in high-risk patients with ACS.^[16]^ There are several data that support the use of prasugrel/ticagrelor over clopidogrel in patients with ACS and diabetes. The Trial to Assess Improvement in Therapeutic Outcomes by Optimizing Platelet Inhibition with Prasugrel-Thrombolysis in Myocardial Infarction (TRITON-TIMI) 38 and The Targeted Platelet Inhibition to Clarify the Optimal Strategy to Medically Manage Acute Coronary Syndromes (TRILOGY ACS) were compared the prasugrel with the clopidogrel. 6690 diabetic patients have compared the composite primary endpoint of cardiovascular death, non-fatal MI, and cerebrovascular accident (CVA). The overall result was in favor of prasugrel (relative reduction; 0.80, 95% CI; [0.66–0.97]).^[17,18]^ There is diabetes subgroup data from the TRITON-TIMI 38; MI was also reduced in the prasugrel group (HR, 0.82; *P* = .006).^[19]^ Although the bleeding complications of prasugrel were higher in TRITON-TIMI 38, the study of the subgroup patients (core clinical cohort) with no history of stroke/transient ischemic attack, age <75 years, and weight ≥60 kg had substantial decreases in the ischemic event with prasugrel compared with clopidogrel without increasing TIMI major bleeding.^[20]^ In the platelet inhibition and patient outcomes trial, ticagrelor and clopidogrel were compared in patients with ACS and diabetes. Although the diabetes patient cohort results did not reduce the composite primary endpoint of CV death, non-fatal MI or non-fatal CVA, ticagrelor reduced composite primary endpoint in patients with HbA1c above the median (HbA1c 6.0%).^[21]^ Although these data have shown the clinical benefit of prasugrel/ticagrelor over clopidogrel in patients with ACS and diabetes, recent guideline pointed out that there is no convincing evidence that the presence of diabetes should affect decision making with respect to the choice of prasugrel and ticagrelor.^[9]^ This opinion would be caused by the increase of major bleeding events in prasugrel and ticagrelor groups.

Even though many studies of prasugrel and ticagrelor reported to reduce ischemic events and mortality, our study showed that prasugrel/ticagrelor increased major bleeding events without improvement of the composite of CD, recurrent MI or stroke. There are several studies similar to our result, particularly in studies of East Asian patients. Goto et al^[6]^ evaluated the ticagrelor and clopidogrel in Japanese, Taiwanese, and South Korean patients with ACS. This study included 34.5% of diabetes patients. The major bleeding events were higher, albeit not significantly, in ticagrelor-treated patients and the composite of cardiovascular death, MI or stroke was not different between ticagrelor and clopidogrel group. Park et al^[8]^ compared the ticagrelor with clopidogrel in Korean patients with AMI. This study included diabetes patient about 22%; ticagrelor did not reduce the composite of CD, non-fatal MI or stroke at 6 month (odds ratio [OR]; 0.784, 95% CI; [0.491–1.253]) and was associated with increased risk of in-hospital TIMI major bleeding (OR; 1.971, 95% CI; [1.086–3.577], *P*-value = .026). They compared the prasugrel with clopidogrel as the same method; prasugrel did not also reduce the composite of CD, non-fatal MI or stroke at 6 months (OR; 0.998, 95% CI; [0.459–2.171]), but increased the in-hospital TIMI major or minor bleeding (OR; 1.521, 95% CI; [1.028–2.251]).

Pathologically, atherosclerotic plaques in diabetes patients have a character of neovascularization due to angiogenesis of the adventitial vasa vasoum, which may be related with a high risk of intraplaque hemorrhage.^[22]^ From a racial point of view, the East Asian patients are well known to have a lower BMI, and the differences in thrombogenicity, platelet P2Y_12_ receptor inhibition, and propensity for bleeding complication, and therefore tend to show the higher risk of antithrombotic agents related bleeding complications compared with Western patients.^[23–26]^ Tantry et al^[16]^ proposed the following classification based on P2Y_12_ reaction units (PRUs): low on-treatment platelet reactivity (LPR), PRU ≤85; high on-treatment platelet reactivity (HPR), 208 < PRU; and on-treatment platelet reactivity (OPR) within the therapeutic window, 85 < PRU ≤208 OPR within therapeutic window. Lee et al^[5]^ demonstrated that the 10 mg prasugrel has mean PRU value as 83.7, 180 mg ticagrelor as 49.1, and 5 mg prasugrel as 168.5 at 2 to 4 weeks after discharge in East Asian patients with ACS. This data imply that the 10 mg prasugrel and 180 mg ticagrelor would have an LPR in East Asian patients; the use of them could give rise to more increased bleeding complications. In the PRASugrel compared with clopidogrel For Japanese PatIenTs with ACS undergoing PCI (PCIPRASFIT-ACS) study, adjusted-doses of prasugrel (20 of loading dose/3.75 mg of maintenance dose) resulted in lower ischemic events without increased bleeding complications compared to clopidogrel.^[27]^ It suggests that the lower dose of prasugrel/ticagrelor is more effective and safer in East Asian patients. We evaluated the efficacy and safety of prasugrel/ticagrelor in diabetes patient, and diabetes patients have a higher thrombotic risk. However, prasugrel/ticagrelor did not reduce the composite of CD, recurrent MI or stroke, but increased major bleeding. Therefore, we should consider the use of a lower dose of prasugrel/ticagrelor in East Asian patients with MI and diabetes.

There is a report concerning insulin-treated diabetes mellitus (ITDM) patients have greater ADP-induced platelet aggregation, and the cardiovascular event might have increased in ITDM patients.^[28]^ However, there were no significant differences in clinical outcomes between the clopidogrel and the prasugrel/ticagrelor group according to each diabetes medications in subgroup analysis (Supplement table). These results are thought that ITDM patients were only 5.8% in our propensity-matched cohort, which could be underpowered the impact of insulin therapy on the development of cardiovascular events in our data.

This study has several limitations. First, our study is based on a prospective, observational registry, but a small-scale, non-randomized study. Second, although statistical adjustment including propensity score matching was performed, several important variables were still significantly different between the clopidogrel and the prasugrel/ticagrelor group. Third, our study did not evaluate the platelet function tests to assess HPR. Fourth, the incidence of clinical adverse events was relatively lower than previous KAMIR or other random studies.^[19,29,30]^ The reason is that our study excluded the patients discontinued or switched antiplatelet agents, and cause of discontinuing or switching of antiplatelet agent might be associated with the occurrence of clinical adverse events during hospitalization. Therefore, it might have been underestimated the incidence of clinical adverse events in our study compared with other clinical studies including Korean patients with AMI.

## 5 Conclusions

Our study shows that the use of prasugrel/ticagrelor did not improve the composite of CD, recurrent MI or stroke, however, significantly increased major bleeding events in Korean patients with MI and diabetes undergoing PCI. Consequently, prasugrel/ticagrelor should be used more carefully monitored for bleeding complication, especially in patients with low GFR (Ccr <60 ml/min/1.73 m^2^), with hypertension, underwent trans-femoral approach and diagnosed as NSTEMI. The effort to find the optimal dose of antiplatelet agents reaching OPR within the therapeutic window is required in patients with MI and diabetes. Large-scale, long-term, randomized trials should be required to find the efficacy and safety dose of prasugrel/ticagrelor in East Asian patients with MI and diabetes.

## Acknowledgments

We sincerely thank those who participated in data collection and management.

## Author contributions

**Conceptualization:** Kye Taek Ahn, Seok-Woo Seong, Ung Lim Choi, Jin-Ok Jeong.

**Data curation:** Kye Taek Ahn, Seok-Woo Seong, Jin-Ok Jeong.

**Formal analysis:** Kye Taek Ahn, Seok-Woo Seong, Ung Lim Choi.

**Funding acquisition:** Jin-Ok Jeong, Myung Ho Jeong.

**Investigation:** Jin-Ok Jeong, Seon-Ah Jin, Jun Hyung Kim, Jae-Hwan Lee.

**Methodology:** Kye Taek Ahn, Seon-Ah Jin, Jun Hyung Kim, Si Wan Choi.

**Project administration:** Jin-Ok Jeong, Seon-Ah Jin, Jae-Hwan Lee.

**Resources:** Jin-Ok Jeong, Myung Ho Jeong, Shung Chull Chae, Young Jo Kim, Chong Jin Kim, Hyo-Soo Kim, Myeong-Chan Cho, Hyeon-Cheol Gwon, In-Whan Seong.

**Software:** Kye Taek Ahn, Myung Ho Jeong.

**Supervision:** Jin-Ok Jeong, Seon-Ah Jin, Si Wan Choi, Myung Ho Jeong, Shung Chull Chae, Young Jo Kim, Chong Jin Kim, Hyo-Soo Kim, Myeong-Chan Cho, Hyeon-Cheol Gwon, In-Whan Seong.

**Validation:** Kye Taek Ahn.

**Visualization:** Kye Taek Ahn, Si Wan Choi.

**Writing – original draft:** Kye Taek Ahn, Seok-Woo Seong, Ung Lim Choi.

**Writing – review and editing:** Kye Taek Ahn, Jin-Ok Jeong.

Jin-Ok Jeong orcid: 0000-0003-0763-4754.

## Supplementary Material

Supplemental Digital Content
