# Supplementary material for: Comparison of 1-year clinical outcomes between prasugrel and ticagrelor versus clopidogrel in type 2 diabetes patients with acute myocardial infarction underwent successful percutaneous coronary intervention
Source: Medicine (Baltimore). 2019 Mar 15;98(11):e14833. doi: 10.1097/MD.0000000000014833 (PMC6426627; doi:10.1097/MD.0000000000014833)
Supplement: Supplemental Digital Content [file medi-98-e14833-s001.doc]

Supplement Table 1. Subgroup analysis: composite of CD, recurrent MI or stroke in propensity score matching patients

|  | C | P & T | P Value | HR (95% CI) | P Value | P Value for interaction |
| --- | --- | --- | --- | --- | --- | --- |
|  | No. of Events/total (%) | No. of Events/total (%) |  |  |  |  |
| Age (years) |  |  |  |  |  | 0.746 |
| ≥65 | 137/1130 (12.1) | 37/376 (9.8) | 0.264 | 0.799 [0.555-1.148] | 0.225 |  |
| <65 | 78/1236 (6.3) | 34/317 (5.5) | 0.536 | 0.872 [0.583-1.305] | 0.506 |  |
| Gender |  |  |  |  |  | 0.899 |
| Male | 131/1706 (7.7) | 48/768 (6.3) | 0.210 | 0.812 [0.583-1.130] | 0.216 |  |
| Female | 84/660 (12.7) | 23/225 (10.2) | 0.346 | 0.787 [0.496-1.248] | 0.308 |  |
| Ccr (ml/min/1.73m^2^) |  |  |  |  |  | 0.310 |
| <60 | 126/906 (13.9) | 35/271 (12.9) | 0.689 | 0.950 [0.653-1.382] | 0.790 |  |
| ≥60 | 80/1409 (5.7) | 29/703 (4.1) | 0.144 | 0.710 [0.464-1.086] | 0.114 |  |
| Hypertension |  |  |  |  |  | 0.632 |
| Yes | 155/1467 (10.6) | 46/567 (8.1) | 0.098 | 0.758 [0.545-1.053] | 0.099 |  |
| No | 60/899 (6.7) | 25/426 (5.9) | 0.632 | 0.870 [0.546-1.388] | 0.560 |  |
| Puncture |  |  |  |  |  | 0.700 |
| Trasnradial | 52/760 (6.8) | 27/455 (5.9) | 0.551 | 0.844 [0.530-1.343] | 0.473 |  |
| Transfemoral | 156/1548 (10.1) | 39/515 (7.6) | 0.099 | 0.757 [0.533-1.075] | 0.119 |  |
| Clinical presentation |  |  |  |  |  | 0.799 |
| ST elevation MI | 116/1153 (10.1) | 36/473 (7.6) | 0.134 | 0.753 [0.518-1.095] | 0.137 |  |
| Non ST elevation MI | 99/1213 (8.2) | 35/520 (6.7) | 0328 | 0.804 [0.547-1.183] | 0.269 |  |
| Body weight (kg) |  |  |  |  |  | 0.154 |
| <60 | 76/667 (11.4) | 26/226 (11.5) | 1.000 | 1.002 [0.642-1.565] | 0.992 |  |
| ≥60 | 130/1652 (7.9) | 39/749 (5.2) | 0.020 | 0.655 [0.458-0.937] | 0.021 |  |
| BMI (kg/m^2^) |  |  |  |  |  | 0.303 |
| <25 | 142/1468 (9.7) | 46/559 (8.2) | 0.346 | 0.846 [0.607-1.180] | 0.325 |  |
| ≥25 | 62/846 (7.3) | 19/415 (4.6) | 0.067 | 0.622 [0.372-1.041] | 0.071 |  |
| HbA1C (%) |  |  |  |  |  | 0.654 |
| ≤6.5 | 59/537 (11.0) | 14/200 (7.0) | 0.127 | 0.608 [0.340-1.090] | 0.095 |  |
| >6.5 | 111/1426 (7.8) | 36/644 (5.6) | 0.079 | 0.706 [0.485-1.028] | 0.069 |  |
| DM medication |  |  |  |  |  | 0.610 |
| Oral hypoglycemic agent | 178/1936 (9.2) | 61/846 (7.2) | 0.091 | 0.777 [0.581-1.040] | 0.090 |  |
| Insulin | 14/144 (9.7) | 3/53 (5.7) | 0.414 | 0.562 [0.161-1.956] | 0.365 |  |

CD, cardiac death; MI, myocardial infarction; Ccr, creatinine clearance; BMI, body mass index.

Supplement Table 2. Subgroup analysis: major bleeding in propensity score matching patients

|  | C | P & T | P Value | HR (95% CI) | P Value | P Value for interaction |
| --- | --- | --- | --- | --- | --- | --- |
|  | No. of Events/total (%) | No. of Events/total (%) |  |  |  |  |
| Age (years) |  |  |  |  |  | 0.385 |
| ≥65 | 30/1130 (2.7) | 18/376 (4.8) | 0.044 | 1.754 [0.978-3.146] | 0.060 |  |
| <65 | 17/1236 (1.4) | 10/617 (1.6) | 0.684 | 1.195 [0.542-2.633] | 0.659 |  |
| Gender |  |  |  |  |  | 0.631 |
| Male | 32/1706 (1.9) | 22/768 (2.9) | 0.137 | 1.496 [0.866-2.584] | 0.149 |  |
| Female | 15/660 (2.3) | 6/225 (2.7) | 0.800 | 1.148 [0.445-2.958] | 0.776 |  |
| Ccr (ml/min/1.73m^2^) |  |  |  |  |  | 0.062 |
| <60 | 20/906 (2.2) | 15/271 (5.5) | 0.008 | 2.616 [1.339-5.112] | 0.005 |  |
| ≥60 | 24/1409 (1.7) | 13/703 (1.8) | 0.861 | 0.973 [0.393-2.412] | 0.954 |  |
| Hypertension |  |  |  |  |  | 0.088 |
| Yes | 30/1467 (2.0) | 22/567 (3.9) | 0.020 | 1.915 [1.100-3.333] | 0.022 |  |
| No | 17/899 (1.9) | 6/426 (1.4) | 0.655 | 0.689 [0.272-1.749] | 0.434 |  |
| Puncture |  |  |  |  |  | 0.770 |
| Trasnradial | 10/760 (1.3) | 0/455 (0) | 0.017 | 0.022 [0.000-4.216] | 0.155 |  |
| Transfemoral | 34/1548 (2.2) | 27/515 (5.2) | 0.001 | 2.462 [1.480-4.094] | 0.001 |  |
| Clinical presentation |  |  |  |  |  | 0.064 |
| ST elevation MI | 30/1153 (2.6) | 12/473 (2.5) | 1.000 | 0.973 [0.497-1.907] | 0.937 |  |
| Non ST elevation MI | 17/1213 (1.4) | 16/520 (3.1) | 0.022 | 2.073 [1.047-4.105] | 0.036 |  |
| Body weight (kg) |  |  |  |  |  | 0.835 |
| <60 | 21/667 (3.1) | 12/226 (5.3) | 0.153 | 1.696 [0.834-3.446] | 0.145 |  |
| ≥60 | 23/1652 (1.4) | 16/749 (2.1) | 0.222 | 1.527 [0.802-2.908] | 0.198 |  |
| BMI (kg/m^2^) |  |  |  |  |  | 0.832 |
| <25 | 36/1468 (2.5) | 21/559 (3.8) | 0.132 | 1.490 [0.870-2.552] | 0.147 |  |
| ≥25 | 8/846 (0.9) | 7/415 (1.7) | 0.275 | 1.948 [0.683-5.553] | 0.212 |  |
| HbA1C (%) |  |  |  |  |  | 0.332 |
| ≤6.5 | 13/537 (2.4) | 10/200 (5.0) | 0.093 | 1.882 [0.825-4.297] | 0.133 |  |
| >6.5 | 28/1426 (2.0) | 15/644 (2.3) | 0.618 | 1.143 [0.610-2.140] | 0.676 |  |
| DM medication |  |  |  |  |  | 0.567 |
| Oral hypoglycemic agent | 37/1936 (1.9) | 24/846 (2.8) | 0.158 | 1.449 [0.865-2.430] | 0.159 |  |
| Insulin | 6/144 (4.2) | 2/53 (3.8) | 1.000 | 0.882 [0.178-4.382] | 0.878 |  |

CD, cardiac death; MI, myocardial infarction; Ccr, creatinine clearance; BMI, body mass index.

Supplement Table 3. Subgroup analysis: Composite of CD, recurrent MI, stroke or major bleeding in propensity score matching patients

|  | C | P & T | P Value | HR (95% CI) | P Value | P Value for interaction |
| --- | --- | --- | --- | --- | --- | --- |
|  | No. of Events/total (%) | No. of Events/total (%) |  |  |  |  |
| Age (years) |  |  |  |  |  | 0.815 |
| ≥65 | 160/1130 (14.2) | 53/376 (14.1) | 0.999 | 0.996 [0.730-1.360] | 0.982 |  |
| <65 | 92/1236 (7.4) | 43/617 (7.0) | 0.776 | 0.933 [0.650-1.340] | 0.708 |  |
| Gender |  |  |  |  |  | 0.913 |
| Male | 156/1706 (9.1) | 67/768 (8.7) | 0.762 | 0.953 [0.715-1.269] | 0.740 |  |
| Female | 96/660 (14.5) | 29/225 (12.9) | 0.581 | 0.880 [0.581-1.334] | 0.548 |  |
| Ccr (ml/min/1.73m^2^) |  |  |  |  |  | 0.214 |
| <60 | 141/906 (15.6) | 47/271 (17.3) | 0.508 | 1.145 [0.823-1.593] | 0.421 |  |
| ≥60 | 99/1409 (7.0) | 42/703 (6.0) | 0.405 | 0.838 [0.584-1.203] | 0.338 |  |
| Hypertension |  |  |  |  |  | 0.850 |
| Yes | 179/1467 (12.2) | 65/567 (11.5) | 0.650 | 0.935 [0.704-1.242] | 0.642 |  |
| No | 73/899 (8.1) | 31/426 (7.3) | 0.622 | 0.897 [0.590-1.366] | 0.614 |  |
| Puncture |  |  |  |  |  | 0.180 |
| Trasnradial | 60/760 (7.9) | 27/455 (5.9) | 0.208 | 0.729 [0.463-1.149] | 0.173 |  |
| Transfemoral | 184/1548 (11.9) | 63/515 (12.2) | 0.875 | 1.055 [0.792-1.405] | 0.714 |  |
| Clinical presentation |  |  |  |  |  | 0.287 |
| ST elevation MI | 139/1153 (12.1) | 46/473 (9.7) | 0.197 | 0.798 [0.572-1.114] | 0.185 |  |
| Non ST elevation MI | 113/1213 (9.3) | 50/520 (9.6) | 0.858 | 1.033 [0.740-1.441] | 0.849 |  |
| Body weight (kg) |  |  |  |  |  | 0.080 |
| < 60 | 91/667 (13.6) | 37/226 (16.4) | 0.324 | 1.220 [0.832-1.787] | 0.309 |  |
| ≥60 | 149/1652 (9.0) | 53/749 (7.1) | 0.113 | 0.774 [0.566-1.059] | 0.110 |  |
| BMI (kg/m^2^) |  |  |  |  |  | 0.272 |
| <25 | 168/1468 (11.4) | 64/559 (11.4) | 1.000 | 1.007 [0.755-1.343] | 0.960 |  |
| ≥25 | 70/846 (8.3) | 26/415 (6.3) | 0.216 | 0.743 [0.474-1.166] | 0.196 |  |
| HbA1C (%) |  |  |  |  |  | 0.621 |
| ≤6.5 | 68/537 (12.7) | 24/200 (12.0) | 0.900 | 0.940 [0.590-1.498] | 0.795 |  |
| >6.5 | 133/1426 (9.3) | 49/644 (7.6) | 0.210 | 0.811 [0.585-1.126] | 0.211 |  |
| DM medication |  |  |  |  |  | 0.481 |
| Oral hypoglycemic agent | 206/1936 (10.6) | 83/846 (9.8) | 0.544 | 0.921 [0.714-1.189] | 0.528 |  |
| Insulin | 20/144 (13.9) | 5/53 (9.4) | 0.478 | 0.633 [0.238-1.689] | 0.361 |  |

CD, cardiac death; MI, myocardial infarction; Ccr, creatinine clearance; BMI, body mass index.
